# MINISTOP 3.0: Implementation of a mHealth obesity prevention program within Swedish child healthcare – study protocol for a cluster randomized controlled trial

**DOI:** 10.1186/s12889-024-20137-0

**Published:** 2024-09-27

**Authors:** Kristin Thomas, Marie Löf, Maria Lundgren, Maria Fagerström, Kylie D. Hesketh, Vicki Brown, Henrike Häbel, Christine Delisle Nyström

**Affiliations:** 1 Department of Medicine, Huddinge, Karolinska Institutet, Neo Huddinge, 14183 Sweden; 2 Department of Health, Medicine and Caring Sciences, Division of Society and Health, Linköping University, Linköping, 58183 Sweden; 3 Institute for Physical Activity and Nutrition (IPAN), Deakin University, Geelong, 3125 Australia; 4 Institute for Health Transformation (IHT), Deakin Health Economics, Deakin University, Geelong, 3125 Australia

**Keywords:** Cost-effectiveness, Effectiveness, Implementation, Lifestyle behaviours, Obesity prevention, Pre-school children

## Abstract

**Background:**

Previously, we have reported on the efficacy and real-world effectiveness of a parent-oriented mobile health intervention (MINISTOP 1.0 and 2.0), which have shown improvements in pre-school children’s lifestyle behaviours. However, there is a need for implementation evidence. The overall aims of this study are to: (i) compare two different implementation strategies for MINISTOP 3.0 (Basic vs. Enhanced) on: acceptability, appropriateness, feasibility, organizational readiness to implement MINISTOP 3.0 within Swedish child healthcare (primary outcomes) as well as reach, costs, and adoption of MINISTOP 3.0 (secondary outcomes); (ii) evaluate cost-effectiveness of MINISTOP 3.0; (iii) explore the sustainability of MINISTOP 3.0; (iv) evaluate the determinants of effectiveness of MINISTOP 3.0 on children’s key lifestyle behaviours; and (v) investigate the long-term effects of MINISTOP 3.0 on children’s body mass index.

**Methods:**

A hybrid type III implementation-effectiveness design will be used. A cluster randomized controlled trial will be conducted to compare the effects of basic versus enhanced implementation strategies on the outcomes at the child healthcare level. A minimum of 50 child healthcare centers across Sweden will participate and we aim to recruit 120 nurses. Child healthcare nurses in both groups will offer the MINISTOP 3.0 app to the families at the 2.5/3-year routine visit. Basic implementation strategies include educational meeting with nurses, formal implementation blueprint, develop/distribute educational materials and enhanced implementation includes all aforementioned strategies plus auditing/providing feedback and ongoing training for nurses. All outcomes will be assessed at baseline and 12 months post-implementation. Implementation outcomes will be assessed quantitatively using questionnaires and sustainability will be assessed qualitatively at 12 months. Children’s key lifestyle behaviours will be collected through a parental questionnaire within the MINISTOP app at baseline and 6 months after they have received the app. Children’s weight/height will be measured at routine visits at 2.5/3 (baseline), 4 and 5 years of age.

**Discussion:**

This study will provide important implementation evidence with regards to implementing mHealth interventions within Swedish child healthcare at scale and these results have the potential to be generalized to other digital interventions being implemented in child healthcare.

**Trial registration:**

ClinicalTrials.gov, NCT05667753. Registered December 29, 2022.

**Supplementary Information:**

The online version contains supplementary material available at 10.1186/s12889-024-20137-0.

## Background

Childhood obesity is a major public health challenge with approximately 39 million children under five years of age being classified as overweight or obese in 2020 [1]. In Sweden the prevalence of overweight and obesity in four-year-olds has remained relatively stagnant for the past 12 years [2]. However, in a recent study utilizing the national quality register for child healthcare for three Swedish regions (Dalarna, Jönköping, and Sörmland) a significant increase in the prevalence of overweight and obesity in four-year-olds from before to during the COVID-19 pandemic was observed [3]. Similar findings were also found in Region Stockholm in 2022, where an increase in the prevalence of overweight and obesity in four-year-olds was found, where social isolation in combination with changes in eating patterns, decreased physical activity, and increased screen time being deemed the root causes [2]. As the majority of weight gain in adolescents with overweight and obesity has been found to occur between the ages of two and six years [4], this demonstrates the importance of scalable obesity prevention interventions in the pre-school years.

The Mobile-based Intervention Intended to Stop Obesity in Preschoolers (MINISTOP) is a promising obesity prevention program with the overall aim to support parents of pre-school aged children to create healthy lifestyle behaviours (PI: Marie Löf) [5, 6]. In 2014–15 the efficacy of MINISTOP was evaluated in a randomized controlled trial in 315 4-year-old children using accurate and objective methodologies. For the primary outcome, fat mass index, no statistically significant difference was observed between the intervention and control groups; however, for a composite score including both dietary and physical activity variables, children in the intervention group had a 99% higher odds of increasing their health behaviour composite score compared to their counterparts in the control group (*p* = 0.008) [5]. MINISTOP 1.0 was delivered under controlled conditions by researchers, which is not scalable into real-world settings. Therefore, in 2019–22 a hybrid type I implementation-effectiveness trial was conducted evaluating MINISTOP when delivered by child healthcare nurses through child healthcare at routine visits (*n* = 552). In this study the MINISTOP app was available in four of the most common languages spoken in Sweden (Swedish, English, Arabic, and Somali). Compared to the control group, children in the intervention group reported lower intakes of sweet and savoury treats (-7 g/day, *p* = 0.001), sweet drinks (-32 g/day, *p* < 0.001), and screen time (-7 min/day, *p* = 0.012) post-intervention. Furthermore, post-intervention parents in the intervention group reported statistically significant higher parental self-efficacy scores for promoting healthy diet (*p* = 0.008) and physical activity behaviours (*p* = 0.009) in their children [6].

In Sweden, child healthcare is provided to all children from birth to five years of age free of charge. As child healthcare reaches almost all children living in Sweden regardless of socioeconomic position or migrant status [7], this provides an ideal arena to integrate an obesity prevention intervention to support parents to promote healthy lifestyle behaviours in their children. Furthermore, interventions that can be incorporated into existing systems have a higher chance of success compared to interventions that are more resource intensive [8]. As MINISTOP is a mobile health (mHealth) intervention and takes less than ten minutes to register and introduce parents to the app at routine visits within child healthcare, it has potential to be integrated into routine care. Following the MINISTOP 2.0 trial there was great interest from child healthcare to implement MINISTOP into routine care at scale, enabling research regarding implementation in real-world conditions.

Finally, there is a need for implementation evidence including information on acceptability, feasibility, sustainability, reach, effectiveness, and cost-effectiveness to inform key stakeholders making decisions on whether and how new programs could be implemented into healthcare. According to the National Institute of Health implementation is a key component of the translation process and it is an essential pre-requisite in order for research to yield improvements in public health [9]. As a lot of successful and promising research does not transition into practice [9], this demonstrates the need for large-scale implementation-effectiveness studies.

## Aims

The overall aim of this study protocol is to describe the methodology of the MINISTOP 3.0 trial. A cluster randomized controlled trial utilizing a hybrid type III implementation-effectiveness trial design is being used to compare two different implementation strategies (Basic vs. Enhanced) when implementing MINISTOP 3.0 at scale (objectives one to three). Furthermore, data on children’s lifestyle behaviours and anthropometrics will be passively collected through the MINISTOP 3.0 app and health journals (objectives four and five). The specific objectives are:

(i) To compare two different implementation strategies for MINISTOP 3.0 (i.e., Basic vs. Enhanced) on:
  - acceptability, appropriateness, feasibility, and organizational readiness to implement MINISTOP 3.0 within child healthcare (primary outcomes).
  - reach, costs, and adoption of MINISTOP 3.0 within child healthcare (secondary outcomes).
(ii) To evaluate the cost-effectiveness of implementing MINISTOP 3.0.
(iii) To explore the sustainability of MINISTOP 3.0 within child healthcare (secondary outcome).
(iv) To evaluate the determinants of effectiveness of MINISTOP 3.0 (e.g., usage, language) on children’s key dietary indicators, physical activity, and screen time (secondary outcomes).
(v) To investigate the long-term effects of MINISTOP 3.0 on children’s body mass index (BMI) at four and five years of age.

## Methods

### Study design, recruitment, and participants

This project utilizes a hybrid type III implementation-effectiveness trial design as the primary focus is testing two different implementation strategies [10]. A cluster randomized controlled trial is being used to compare two different implementation strategies for MINISTOP (Basic vs. Enhanced). A minimum of 50 child healthcare centers across Sweden will be randomly allocated to either the basic implementation group or the enhanced implementation group. All child healthcare nurses at the participating centers will be provided with oral and written information from the research team regarding the study. If they agree to participate, they will provide digital informed consent through signing with BankID (a secure digital signature) and thereafter will fill in the baseline questionnaire (digital). To be included in the study nurses need to be employed at one of the participating child healthcare centers. Nurses were excluded if they had worked at a center for less than three months as they do not have the ability to rate or appraise workplaces readiness.

At the routine 2.5/3-year visit at child healthcare all families will be offered the MINISTOP 3.0 app by their nurse as part of routine care. Using the MINISTOP 3.0 control tower (i.e., the child healthcare interface), nurses will register the participating parent for the MINISTOP 3.0 app using their personal identity number and telephone number. The parent will then receive a link via SMS where they can download MINISTOP. Once downloaded, they sign into MINISTOP using their BankID. Thereafter, they will receive a push notification within the MINISTOP 3.0 app asking them to participate in a study on their child’s diet, physical activity, and screen time behaviours. Within this push notification there is a link for the full study information and if the parent agrees to participate, they will consent digitally using their BankID. Thereafter, they will fill in the baseline questionnaire.

A mixed methods approach will be used to collect data at the organizational level (i.e., child healthcare) as well as the individual level (i.e., child healthcare nurses, parents, and children). The `Implementation Outcomes´ [11] and the `RE-AIM´ [12] evaluation frameworks will be utilized to identify relevant implementation and effectiveness outcomes.

**Fig. 1:**
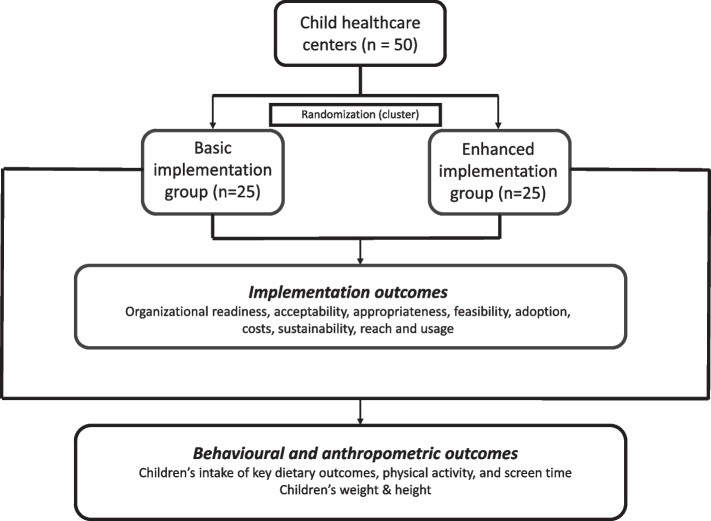
Flowchart for MINISTOP 3.0

**Fig. 2:**
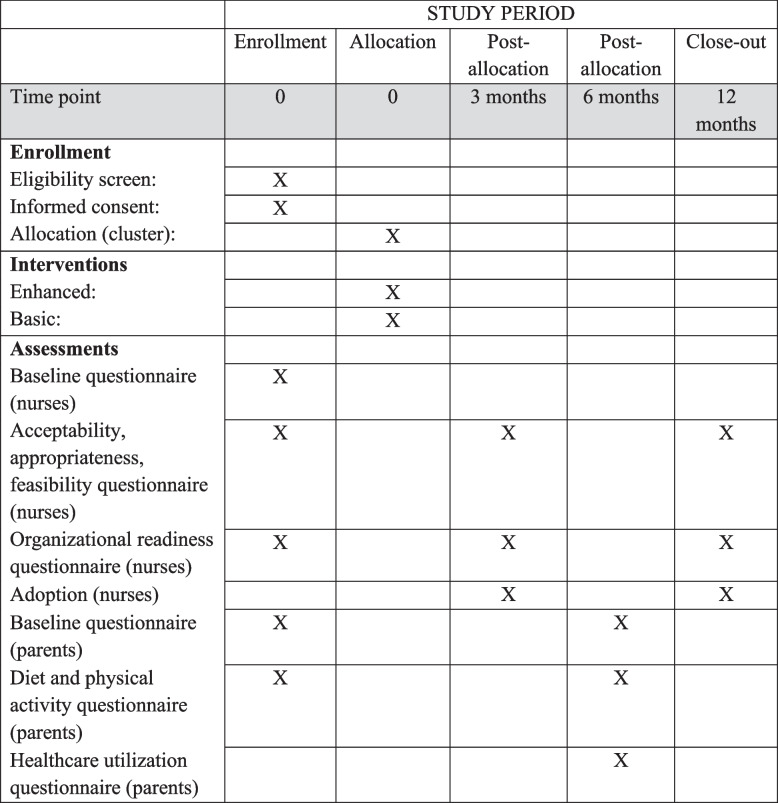
Participant timeline (SPIRIT)

This study is reported according to the SPIRIT 2013 statement [13]. The flowchart and participant timeline for the MINISTOP 3.0 trial is shown in Figs. 1 and 2, respectively.

### Randomization and blinding

Participating child healthcare centers will start in waves. Start dates for the waves are decided upon by the research team and the in-house project leader at child healthcare. The in-house project leader will communicate these start dates to the regional bosses within child healthcare and child healthcare centers can choose a date that suits their center. The participating child healthcare centers will be randomly allocated to either the basic or enhanced implementation group using computer-generated random assignment by an independent statistician. In each wave, the randomization of child healthcare centers will be stratified by center size (i.e., number of nurses) as well as geographical location (i.e., region in Sweden) to ensure balance. Due to the nature of the trial, child healthcare nurses and the research team cannot be blinded.

### MINISTOP 3.0 intervention

**Fig. 3:**
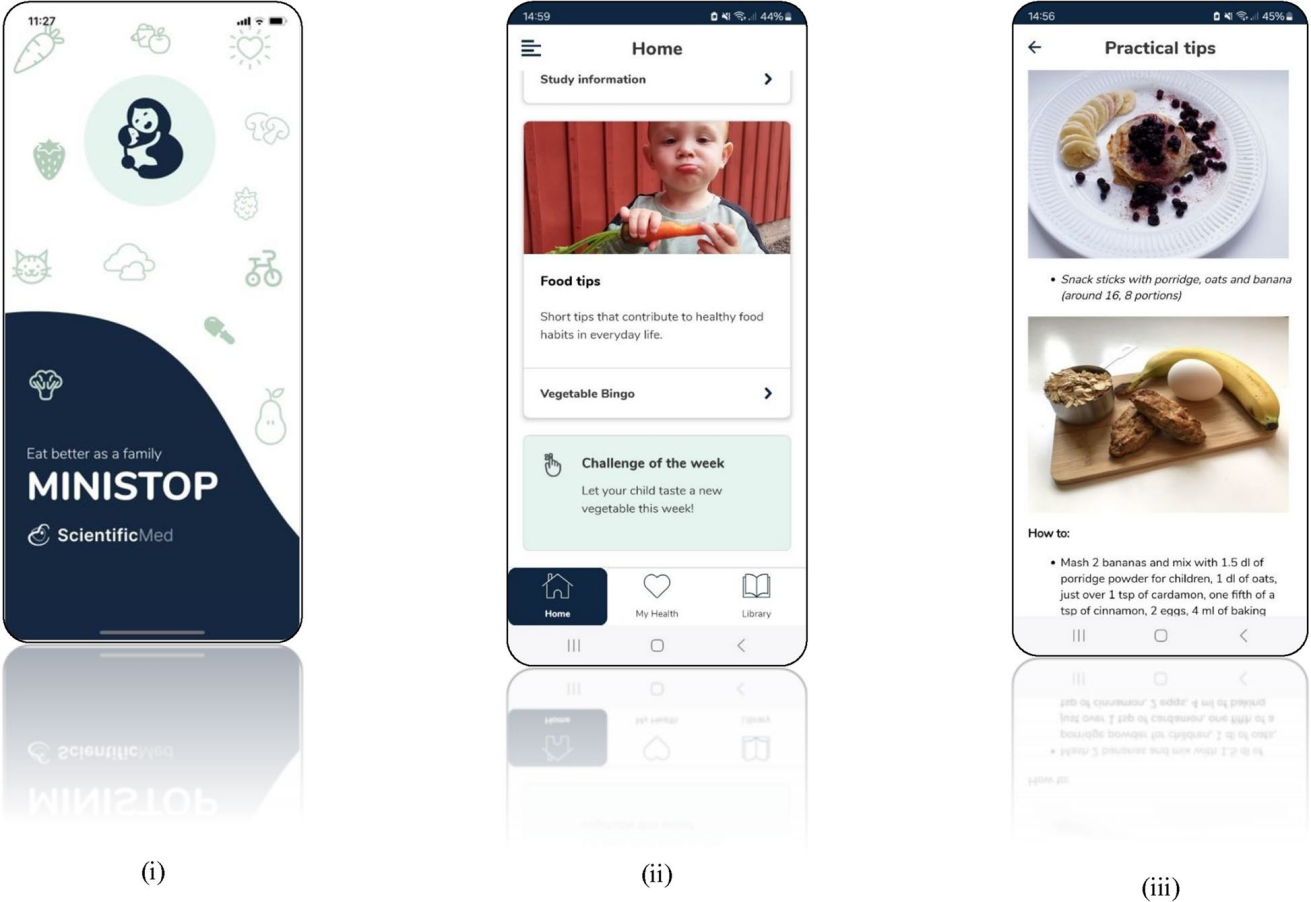
Screen shots of the MINISTOP 3.0 app with the overall aim to prevent overweight and obesity through the promotion of healthy lifestyle behaviours in pre-school aged children. (i) App icon for the MINISTOP 3.0 app. (ii) Home screen of the MINISTOP 3.0 app. (iii) an example of a practical tip in the MINISTOP app

The MINISTOP 3.0 app is a six-month mHealth intervention targeting parents of pre-school aged children. The overall aim of the app is to improve lifestyle behaviours (i.e., diet, physical activity, and sedentary behaviours) in two- to three-year-old children. Screen shots of the MINISTOP 3.0 app are shown in Fig. 3 and the app is available in Swedish, English, Somali, and Arabic.

The MINISTOP 3.0 app builds upon the MINISTOP 1.0 and 2.0 versions contents [14, 15]; however, it utilizes a new technical platform. Similar to the previous versions of the app it is compatible with both Android and IOS. The content of MINISTOP 3.0 is built upon evidence-based recommendations for healthy eating [16], physical activity [17, 18], and screen time [17, 18] for pre-school aged children. The app is based in social cognitive theory [19] as well as important behavioural change techniques (e.g., identification of barriers, shaping knowledge, goal setting, self-monitoring/feedback) [20]. Similar to the MINISTOP 2.0 app, the MINISTOP 3.0’s content is built around thirteen themes (everyday food, breakfast, healthy snacks, physical activity and screen time, sweets and snacks, fruit and vegetables, beverages, snacking, fast food, sleep, meals outside the home, food as a reward/on special occasions, and dental health), with a new theme being introduced every second week [15]. Each theme has three sub-themes with basic facts, practical tips, and strategies that are relevant to that topic. Furthermore, it is important to highlight that the MINISTOP 3.0 app includes information as well as tips and advice for all types of diets (e.g., omnivore, vegetarian, and vegan).

Briefly, within the app there is also a library where parents can access more information and receive practical tips/suggestions on lifestyle behaviours. For instance, there are films by a dietician answering commonly asked questions, recipes for healthy meals and snacks, weekly menus (including grocery lists), ideas/suggestions on how to increase children’s physical activity (indoor and outdoor activities), and a list of in season fruits and vegetables in Sweden by month. Finally, within the app there is a self-monitoring and feedback feature where parents can register their child’s fruit, vegetable, sweet snack, and savoury snack intakes, as well as their physical activity and screen time. At the end of every week parents will get visual feedback (bar graph) as well as a feedback message for the parameters that they registered that week in the app.

After the completion of the MINISTOP 2.0 trial, and based on parental feedback on the app, we have now added craft ideas/tips, a list of in season fruits and vegetables to help families eat more sustainably, 50 new recipes, and 15 videos with a dietician answering commonly asked questions to the MINISTOP 3.0 library. Furthermore, within the app we have also added a weekly challenge which parents can do together with their child. An example of a weekly challenge is, `in the library you can find a list of Swedish fruits and vegetables that are in season, can you include three in season fruits or vegetables in your cooking/snacks this week?´.

### Basic and enhanced implementation groups

The child healthcare centers allocated to the basic implementation group will receive the start-up educational meeting, have the formal implementation blueprint communicated to them, and receive the MINISTOP educational material. The enhanced implementation group will receive the basic package, plus seven monthly update newsletters and two on-going training sessions regarding the MINISTOP 3.0 app and using it in routine practice.

#### Implementation strategies

All implementation strategies are reported according to the ERIC taxonomy [21]. Table 1 provides an overview of the specifications of the implementation strategies utilized in this study.

**Table 1:** Specification of implementation strategies including actor(s), action, action target, dose and timing

Implementation strategy	Actors(s)	Action	Action target	Dose	Timing	Group
Conduct educational meeting^a^ – “Start-up meeting”	Research team, representative from the development team for MINISTOP platform, in-house project leader from child healthcare	Inform and educate on the MINISTOP platform (app and interface), previous scientific evaluations, hands-on guidance how to use and implement MINISTOP in daily routines	Increase knowledge and motivation among child healthcare nurses	Once	Before implementation of MINISTOP commences	Basic & Enhanced
Formal implementation blueprint^b^—Communicated during the start-up meeting	Research team and representatives from child healthcare	A formal implementation blueprint for the MINISTOP implementation was created	To clarify implementation strategy and goals	Once	Before implementation of MINISTOP commences	Basic & Enhanced
Develop and distribute educational materials^c^	Research team	Resources for child healthcare nurses as well as material for the child healthcare nurses to distribute to the families about the MINISTOP app	Increase knowledge about MINISTOP and confidence to use MINISTOP among child healthcare nurses	Once	Before implementation commences	Basic & Enhanced
Audit and provide feedback^d^	Research team	A monthly newsletter where implementation data is collected, summarized and communicated to child healthcare nurses	Increase motivation among child healthcare nurses	Monthly (7 times in total)	Start from 6 months and onwards	Enhanced
Conduct ongoing training^e^ “Online and in-person workshops”	Research team and child health care nurses	Provide training on the MINISTOP concept, hands-on training, discussions, and exercises	Increase confidence and change practice among child healthcare nurses. Facilitate knowledge sharing between colleagues	Twice (one in-person and one online)	At 5 and 10 months	Enhanced

^a^ Strategy 15 ERIC taxonomy

^b^ Strategy 23 ERIC taxonomy

^c^ Strategies 29 and 31 ERIC taxonomy

^d^ Strategy 5 ERIC taxonomy

^e^ Strategy 19 ERIC taxonomy [21]

#### Educational meeting (both implementation groups)

Before the start of each wave a one-hour educational meeting will be conducted with all child healthcare nurses from the participating child healthcare centers. This meeting will be led by members of the research team, the MINISTOP platform developer, and the in-house project leader from child healthcare. This meeting starts with an introduction of what the MINISTOP 3.0 app is, the research behind the MINISTOP app, and how the MINISTOP 3.0 app can be incorporated into routine care at the 2.5/3-year visit. Thereafter, the nurses will be shown how to add families into the control tower in order to provide families with access to the MINISTOP 3.0 app. Finally, the nurses will be provided with information regarding the research study and informed that they will be receiving an email from a member of the research team the next day with full study information and a link for the baseline questionnaire. The meeting will end with an open discussion where all questions from the nurses are addressed.

#### Formal implementation blueprint (both groups)

A formal implementation blueprint was created by members of the research team together with senior representatives from child healthcare. This blueprint was developed before the implementation of MINISTOP commenced and is updated throughout the implementation period. This document contains: (i) the specific aims regarding the implementation of MINISTOP in child healthcare; (ii) the scope of the change (i.e., how nurses will change their routines to incorporate MINISTOP at the 2.5/3 year visit; and (iii) a timeframe of the implementation of MINISTOP including specific milestones. This will be communicated to all child healthcare nurses at the start-up meeting.

#### Develop and distribute educational material (both implementation groups)

Educational material for the nurses will be created by the research team. MINISTOP 3.0 binders will be created and include the following: (i) a one-page pamphlet on the MINISTOP 3.0 app; (ii) a step-by-step manual on how to register families in the MINISTOP 3.0 control tower; (iii) a information booklet on MINISTOP 3.0 that the nurses can show the parents at the 2.5/3-year visit (in all 4 languages); (iv) a one-page handout to provide the families with, informing them about the MINISTOP 3.0 app (in all 4 languages); (v) visual examples of popular topics from the MINISTOP 3.0 app (e.g., daily fruit and vegetable intake for a 2–4 year old; how much sugar is in popular products children consume) that nurses can use to demonstrate the usefulness of the MINISTOP 3.0 app to parents; (vi) laminated QR codes the nurses can have on their desks so parents can easily download the MINISTOP 3.0 app; and (vii) a description of where the nurses can find all of the MINISTOP 3.0 material so that they can download and print more copies when needed. Each center receives one binder, and these binders will be sent via post to all participating child healthcare centers.

#### Audit and provide feedback (enhanced implementation group only)

Personalized monthly newsletters on the center level are sent via email from a member of the research team to child healthcare centers allocated to the enhanced implementation group. These newsletters will start six months after the child healthcare center has implemented MINISTOP 3.0 and will be sent seven times in total. Newsletters will include information on how many families have been added by the specific child healthcare center and in total across all centers that month. Furthermore, information on how many families have been added to each language in the MINISTOP 3.0 app will be provided. The newsletters will also contain information highlighting content in the MINISTOP 3.0 app or tips/advice on strategies on how to introduce MINISTOP 3.0 to the families.

#### Conduct on-going training (enhanced implementation group only)

Each child healthcare center in the enhanced implementation group will receive two on-going training sessions, one 5-months and one approximately 10-months after they started implementing MINISTOP. These sessions will be led by a member of the research team and are one hour in duration. One session will be provided in-person at the child healthcare center and the other session will be conducted digitally. The enhanced sessions are built upon our own study by Fagerström et al. [22], which included MINISTOP, where we investigated nurses, managers, and policy makers perceptions on organizational readiness to implement mHealth interventions in care. In these semi-structured interviews four key areas were identified: (i) capability to manage health-related data; (ii) alignment between mHealth and current organizational ways of working; (iii) governance of mHealth implementation at multiple levels; and (iv) camaraderie within a healthcare team to facilitate use of mHealth in practice [22]. The first enhanced session will incorporate all four aforementioned areas and the session includes: (i) a discussion of how MINISTOP 3.0 data is managed and stored; (ii) a short film by top management sharing their vision regarding the implementation of MINISTOP; (iii) a short film by a nurse on how she uses MINISTOP at the 2.5/3 year routine visit, which facilitates a discussion on how the nurses have integrated MINISTOP into their routines to date; and (iv) a discussion on how they as a unit want to use MINISTOP. The second enhanced session will focus on the alignment between mHealth and current organizational ways of working and camaraderie. This will be done through facilitating a discussion amongst the nurses regarding the challenges and solutions they have encountered while using the MINISTOP 3.0 app to date as well as discussing the value of MINISTOP in their daily work.

## Outcomes

### Implementation outcomes

#### Organizational readiness

The E-READY questionnaire developed by Dannapfel et al. [23] will be used to assess organizational readiness to implement a digital intervention (primary outcome). This questionnaire assesses the perceived readiness for change on both the individual and collective level and consists of 32 questions in six different dimensions rated on a 4-point or 5-point Likert scale. A total sum score will be utilized with a range between 32 and 133 points. Furthermore, we will also use the total score per dimension. All child healthcare nurses will complete this questionnaire at baseline and again 3- and 12-months post-implementation.

#### Acceptability, appropriateness, and feasibility

The questionnaire developed by Weiner et al. [24], which has been shown to have good validity and reliability, will be used to quantitatively assess acceptability, appropriateness, and feasibility of MINISTOP 3.0 within child healthcare (primary outcome). Briefly, this questionnaire consists of four questions for each outcome which are measured on a five-point Likert scale. Child healthcare nurses fill in this questionnaire at baseline and at 3- and 12-months post-implementation.

#### Adoption

Adoption is a secondary outcome and will be assessed at 3-, 8-, and 12-months post-implementation in a sub-set of child healthcare centers that have an online journal tracking system. This will be done using the child healthcare’s online journal system where the number of 2.5/3-year visits will be extracted at 3-months, 8-months, and 12-months post-implementation and compared to the number of families registered in MINISTOP’s control tower.

#### Costs

The costs associated with MINISTOP 3.0 (secondary outcome) will be collected through administrative data from the child healthcare centers regarding the costs of implementing and maintaining the MINISTOP 3.0 platform, as well as the nurses time with the MINISTOP program. Implementation strategies and associated costs will be classified according to sub-categories from the Effective Practice and Organization of Care (EPOC) taxonomy [25] and reported according to published guidance on the economic evaluation of implementation interventions [26].

#### Sustainability

Semi-structured interviews will be conducted (*n* = 15–20) with a purposeful sample of child healthcare nurses who are considered high and low users (based on the MINISTOP 3.0 interface) to explore key factors influencing sustained implementation. All interviews will be carried out at least 12 months after implementation.

#### Reach & usage

Reach and usage are secondary outcomes. The MINISTOP 3.0 interface will be used to assess the number of families being enrolled in each language (i.e., Swedish, English, Arabic, and Somali) to assess reach across cultural groups. Usage of the MINISTOP 3.0 app by parents will be assessed through user data within the app (e.g., how may times they entered the app, the length of the session, how many times they have registered a behaviour etc.).

#### Behavioural outcomes

Children’s lifestyle behaviours (secondary outcomes) are parent reported at baseline and 6-months after they have received the MINISTOP 3.0 app. These are the same questions that have been utilized in the MINISTOP 2.0 trial [6, 15] and the questions regarding diet and physical activity have been adapted for children from the Swedish National Board of Health and Welfare’s survey of health behaviours [27].

#### Diet

Child intake of fruit and vegetables, sweet and savoury treats, and sugar sweetened beverages will be assessed as the average number of standardized portions per day over the past month.

#### Physical activity

The average number of minutes of moderate-to-vigorous physical activity per day over the past month will be reported separately for both weekdays and weekend days.

#### Screen time

The average amount of time per day (minutes) spent in front of a screen (i.e., smartphone, tablet, TV, computer) over the past month will be reported for both weekdays and weekend days.

### Healthcare utilization

Post-intervention parents will be asked to report their child’s healthcare utilization over the past six months. Specifically, they will be asked: (i) have you attended extra appointments with your child healthcare nurse to discuss concerns about your child’s weight, diet, physical activity? (yes or no), if yes how many?; (ii) have you looked for help and/or advice about concerns you had about your child’s weight, diet, or physical activity (yes or no); (iii) for parents that answered yes to question two we would like to know the number of calls/visits to their child healthcare center, family doctor, pediatrician, dietician, or other healthcare professional.

### Anthropometrics

Weight and height are secondary outcomes and will be recorded using standardized procedures at child healthcare at baseline (2.5/3-year visit) and at the 4- and 5-year routine visits. BMI z-scores will be calculated using the values by Cole et al. [28]. Weight and height will be collected for the participating children from their journals. Registry data from all children of similar age residing in participating regions will be used for the comparative group.

### Power calculation

The power calculation is based upon the primary outcome organizational readiness. As this is a relatively new instrument no published studies are available to date. However, unpublished work utilizing this questionnaire in primary care in Sweden showed a normal distribution with a mean of 91.18 points and standard error of 14.82. To test for non-inferiority, the confidence interval method will be applied [29]. A non-inferiority bound of 10% (9-point difference on the scale) was chosen based on previous unpublished data using the E-READY questionnaire. All other primary and secondary implementation outcomes will be tested on superiority of the intervention group (i.e., enhanced implementation) and multiplicity adjusted p-values will be presented.

In a simulation study, a linear mixed effects model was run (1000 repetitions) using the aforementioned mean and standard error as well as assuming an intraclass correlation coefficient of 0.03, 1–3 nurses per child healthcare center (average of two), and 50 child healthcare centers. The model allowed for a random intercept per child healthcare center and was adjusted for wave. With a target power of 80% using a significance level of 0.05, 100 nurses are estimated to be needed. Assuming a 20% drop-out rate 120 nurses will be recruited. R version 4.3.0 [30] was used for the power calculation.

### Data analysis

The intention-to-treat method will be used, including participants in the groups to which they were randomly allocated. The primary analyses will be conducted using multiple imputations, where chained equations [31] will be used to impute missing data for questionnaires. As sensitivity analyses, those with complete data at 12-months, will be conducted.

For the primary outcomes (organizational readiness, acceptability, appropriateness, and feasibility) linear random-effects models will be used to contrast differences between groups at baseline and 12-months post implementation, through specifying the child healthcare center as a random effect to account for clustering, as long as assumptions are not violated. All regression models will be adjusted for the baseline value for the appropriate outcome, nurses age, nurses’ years of experience at current workplace, and wave. Linear random-effects models will also be used to contrast differences between groups for adoption at 3-, 8-, and 12-months post implementation, through specifying the child healthcare center as a random effect to account for clustering, as long as assumptions are not violated. All regression models will be adjusted for the nurses age, nurses’ years of experience at current workplace, and wave. If assumptions for linear regression are violated logistic regression will be used instead.

Descriptive statistics will be used to evaluate reach and parental usage of the MINISTOP 3.0 app. For sustainability, semi-structured interviews will be audio recorded and transcribed verbatim. Finally, a healthcare funder perspective will be used for the cost data analysis in order to assess costs related to both implementation strategies. Total cost, cost per child healthcare center, and cost per child will be reported. Costs will be estimated using administrative data and published costs, and costs will be valued in Swedish Krona for the appropriate reference year. Cost-consequence analysis will be presented, which allow for itemised description of both the costs and outcomes by basic and enhanced implementation strategy.

With regards to the behavioural outcomes and BMI (e.g., z-scores) multivariable linear or logistic regression models will be used depending on the data’s distribution. The child healthcare center will be specified as a random effect to account for clustering. Furthermore, where appropriate regression models will be adjusted for the baseline value for the outcome as well as the child’s age and sex at baseline.

### Ethics approval

This study has been approved by the Swedish Ethical Review Authority (Dnr: 2022–01710-01; 2022–06749-02; 2023–01001-02; 2023–07673-02). Informed consent will be obtained from all participating nurses and parents.

### Trial status

Recruitment has been initiated and is currently ongoing.

## Discussion

The Swedish National Board of Health and Welfare has identified that there is a need for more material, education, and working methods for promoting healthy lifestyle behaviours within child healthcare [7]. Furthermore, lack of time has been identified as one of the main barriers for child healthcare nurses to provide parents with information regarding lifestyle behaviours [32]. Thus, there is a need for a scalable and cost-effective digital tool such as MINISTOP that can support both parents and child healthcare nurses with regards to promoting healthy lifestyle behaviours in pre-school aged children that can be implemented at scale. A unique aspect of MINISTOP is that is has been rigorously evaluated in two previous trials [5, 6]; however, before it can be implemented at scale there is a need for implementation evidence.

In Sweden, health talks and guidance on promoting healthy lifestyle behaviours are part of routine care from three months of age [33]. There is an array of material that is available for nurses to use to support these health talks (e.g., [34, 35]) at routine visits. Health talks at child healthcare initiate the idea of creating healthy lifestyle behaviours with families. In an qualitative study by Alexandrou et al. [36] Swedish child healthcare nurses stated that routine visits are short and that lifestyle behaviours are not possible to change after one conversation. Thus, more support in the home environment is needed to support families in creating healthy lifestyle behaviours, through complementing the messages that they are receiving at child healthcare. MINISTOP has been shown to be effective in promoting healthy lifestyle behaviours in young children [5, 6] and thus is an ideal complement to routine child healthcare. However, before MINISTOP can be implemented at scale, there is a need for implementation evidence in order for key stakeholders to make informed decisions. In this trial we aim to demonstrate non-inferiority between the two implementation strategies (i.e., basic vs. enhanced). As is common in healthcare lack of time has been identified as a major barrier for Swedish child healthcare nurses [32], thus there is a need to find the most efficient and cost-effective strategies for implementation.

A major strength of this study is the type III hybrid design which allows us to concurrently evaluate both implementation and behavioural outcomes. This is important as a hybrid design has the capability to increase the translation of the study findings into routine clinical practice [10]. This study is further strengthened through its randomized controlled trial design. Additionally, the MINISTOP 3.0 trial is the result of more than ten years of research. The first version of the app (MINISTOP 1.0) [14] was well-anchored in behaviour change techniques [20] and social cognitive theory [19] and was evaluated in a randomized controlled trial. Thereafter the app was refined and culturally adapted based on qualitative studies with end-users (i.e., child healthcare nurses and parents with different cultural backgrounds) into a second version, MINISTOP 2.0 (available in Swedish, English, Somali, and Arabic) [37]. The MINISTOP 2.0 app was evaluated in a type I hybrid implementation-effectiveness trial, focusing on effectiveness which was delivered through child healthcare [6, 15]. Based on the feedback from end-users from the MINISTOP 2.0 trial (qualitative sub-study) [36] we made further refinements in order to even better align to the needs of our target population. Consequently, the MINISTOP 3.0 app has undergone rigorous development also including co-creation with end-users. Indeed, the use of co-creation is very important as a systematic review by Halvorsrud et al. [38] found that co-creation improves health-promoting behaviours. Furthermore, the two sessions that the enhanced groups receive are based upon a qualitative study conducted by Fagerström et al. [22] investigating child healthcare nurses perspectives regarding organizational readiness to implement the MINISTOP app within child healthcare. The long study period spanning 12 months is another strength of this study as it allows for us to investigate implementation in real-life over a long period of time. Finally, this study is further strengthened through the exploration of sustainability among both high and low users.

To the best of our knowledge there are no objective measures of organizational readiness, acceptability, appropriateness, and feasibility, which is a limitation of this study. However, it is important to highlight that that implementation in the current study is investigated in a comprehensive manner. Furthermore, as we did not want to overburden the participants, we are unable to investigate the causal mechanisms regarding how the implementation strategies worked. This could be a topic for further research.

## Conclusion

Overall, this study will provide important implementation evidence with regards to implementing a mHealth intervention, MINISTOP 3.0, within Swedish child healthcare and these results have the potential to be generalized to other digital interventions being implemented in healthcare.

## Supplementary Information

Supplementary Material 1.

## Data Availability

Not applicable as this article does not present data.

## Abbreviations

BMI: Body mass index
mHealth: Mobile health
MINISTOP: Mobile based intervention intended to stop obesity in preschoolers
PI: Principal investigator

## Data availability

No datasets were generated or analysed during the current study.
